# *BRAF*^non-V600E^ more frequently co-occurs with *IDH1/2* mutations in adult patients with gliomas than in patients harboring *BRAF*^V600E^ but without a survival advantage

**DOI:** 10.1186/s12883-021-02224-6

**Published:** 2021-05-12

**Authors:** Wei Wang, Maode Wang, Haitao Jiang, Tuo Wang, Rong Da

**Affiliations:** 1 Department of Neurosurgery, The First Affiliated Hospital of Xi’an Jiaotong University, Xi’an, China; 2 Department of Clinical Laboratory, The First Affiliated Hospital of Xi’an Jiaotong University, No.277 Yanta West Road, Xi’an, 710061 Shaanxi China

**Keywords:** Adult patient with glioma, *BRAF*^non-V600E^, *BRAF*^V600E^, *IDH1/2*

## Abstract

**Background:**

The effects of *BRAF*^non-V600E^ and *BRAF*^V600E^ on the outcomes and the molecular characteristics of adult glioma patients are unknown and need to be explored, although *BRAF*^V600E^ has been extensively studied in pediatric glioma.

**Methods:**

Co-occurring mutations and copy number alterations of associated genes in the MAPK and p53 pathways were investigated using data from The Cancer Genome Atlas (TCGA) public database retrieved by cBioPortal. The prognosis of available adult glioma cohorts with *BRAF*^V600E^ and *BRAF*^non-V600E^ mutations were also investigated.

**Results:**

Ninety patients with *BRAF*^V600E^ or *BRAF*^non-V600E^ were enrolled in this study, and data from 52 nonredundant patients were investigated. Glioblastoma multiform was the most common cancer type, with *BRAF* ^non-V600E^ and *BRAF*^V600E^. *TP53* (56.00% vs. 7.41%), *IDH1/2* (36.00% vs. 3.70%), and *ATRX* (32.00% vs. 7.41%) exhibited more mutations in *BRAF*^non-V600E^ than in *BRAF*^V600E^, and *TP53* was an independent risk factor (56.00% vs. 7.41%). Both *BRAF*^non-V600E^ and *BRAF*^V600E^ frequently overlapped with *CDKN2A*/*2B* homozygous deletions (HDs), but there was no significant difference. Survival analysis showed no difference between the *BRAF* ^non-V600E^ and *BRAF*^V600E^ cohorts, even after excluding the survival benefit of *IDH1/2* mutations and considering the *BRAF*^non-V600E^ mutations in the glycine-rich loop (G-loop) and in the activation segment. The estimated mean survival of patients with *BRAF*^non-V600E^ & *IDH1/2*^WT^ with mutations in the G-loop groups was the shortest.

**Conclusions:**

*BRAF*^non-V600E^ exhibited a stronger association with *IDH1/2* mutations than *BRAF*^V600E^, but no survival advantage was found.

**Supplementary Information:**

The online version contains supplementary material available at 10.1186/s12883-021-02224-6.

## Background

*BRAF* (v-raf murine sarcoma viral oncogene homolog B1) is a serine-threonine kinase in the *Ras/Raf/*mitogen-activated protein kinase (MAPK) pathway [1, 2] that transduces mitogenic stimuli after the activation by growth factor receptors that are involved in cell survival, proliferation, and differentiation [3]. MAPK pathway activation is common in various neoplasms. Active *RAS* mutations have been detected in approximately 15% of malignant human tumors.

Compared with *ARAF* and *RAF1*, *BRAF* plays a critical role in kinase activity [4]. A previous study showed that *RAF1* is activated by *BRAF* through direct interactions between proteins and phosphorylation [5]. *BRAF* participates in the pathological mechanism of 7% of human neoplasms, especially in patients with melanoma and colorectal, thyroid, and lung cancer [6, 7]. The expression of *BRAF* is highly restrained [1, 8]. The high expression of *BRAF* in neural cells indicates that it is a vital MEK kinase in neuronal tissues [9, 10]. *BRAF* mutations are found in some central nervous system neoplasms. In pediatric low-grade gliomas (LGGs), these alterations correlate with oncogenic senescence, which may contribute to an improved prognosis [11]. The *BRAF*^V600E^ mutation is rare in adult LGGs and glioblastomas and can only be found in 1 to 5% of samples [12, 13]. While *BRAF* activation contributes to tumor development and progression in the neural stem cells and progenitor cells of *Homo sapiens*, *BRAF* mutations are detected in adult diffuse gliomas and are associated with poor outcomes [14].

Most studies have focused on the *BRAF*^V600E^ mutation, although more than 70 *BRAF* mutations have been reported to date. Mutations in *BRAF* at V600 can activate ERK, which plays a critical role in the G1/S transition by adjusting the expression of cyclin D, cyclin E, and p21Cip1 [15]. The *BRAF*^V600E^ mutation is the most potent MAPK pathway activator, whereas *BRAF*^non-V600E^ mutations are low-activity kinases that slightly stimulate the MAPK pathway [16]. However, these low-activity *BRAF* mutants could activate MAPK signaling in COS-1 cells to a high level by activating *RAF1* [16].

Isocitrate dehydrogenase (IDH) is a frequent mutation associated with a survival benefit in glioma patients and it has been defined as a molecular parameter to define the categories of brain tumors in the updated 2016 edition of the World Health Organization (WHO) Classification of Tumors of the Central Nervous System (CNS) [17]. *IDH1* and *BRAF*^V600E^ mutations are associated with infiltrative gliomas or circumscribed gliomas and glioneuronal tumors, respectively [18, 19], and they are exclusive in most cases [20]. The exact effect of *BRAF* ^non-V600E^ and *BRAF*^V600E^ on the prognosis of glioma patients and whether there are unique molecular characteristics in their MAPK and p53 pathways remain largely unknown.

In this study, co-occurring mutations and copy number alterations of 35 associated genes in the MAPK and p53 pathways were retrieved and investigated, and the prognosis of the available adult glioma cohorts with *BRAF*^V600E^ and *BRAF*^non-V600E^ were evaluated by using The Cancer Genome Atlas (TCGA) public database. We determined that *BRAF*^non-V600E^ exhibited a stronger association with the *IDH1/2* mutation than *BRAF*^V600E^, but no survival advantage was found.

## Methods

### Data collection and enrollment

All data were collected and generated from the TCGA public database using the TCGA data mining tool cBioPortal (https://www.cbioportal.org/) [21, 22]. We strictly followed the TCGA publication guidelines (https://www.cancer.gov/about-nci/organization/ccg/research/structural-genomics/tcga/using-tcga/citing-tcga). In multiple patient cohorts of all twenty available CNS/brain studies (6164 samples), the available data were queried, including the gene mutations, copy number alterations, mRNA expression, and protein expression data of patients with *BRAF* gene mutations. In each study, the mutations were selected for genomic profiles. Samples with mutation data were selected for the patient/case set and entered into three groups: (1) General: *Ras*-*Raf*-*MEK*-*ErK*/*JNK* signaling (26 genes), including *KRAS*, *HRAS*, *BRAF*, *RAF1*, *MAP 3 K1*, *MAP 3 K2*, *MAP 3 K3*, *MAP 3 K4*, *MAP 3 K5*, *MAP 2 K1*, *MAP 2 K2*, *MAP 2 K3*, *MAP 2 K4*, *MAP 2 K5*, *MAPK1*, *MAPK3*, *MAPK4*, *MAPK6*, *MAPK7*, *MAPK8*, *MAPK9*, *MAPK12*, *MAPK14*, *DAB2*, *RASSF1*, and *RAB25*; (2) General: p53 signaling (6 genes), including *TP53*, *MDM2*, *MDM4*, *CDKN2A*, *CDKN2B*, and *TP53BP1*; (3) Other frequently mutated genes, including *IDH1*, *IDH2*, and *ATRX*, were then submitted for query. Among the downloadable data files, the available data regarding the mutations, copy number alterations, mRNA expression, and protein expression were downloaded. In the type of genetic alterations across all samples, samples harboring the *BRAF* mutation were chosen. Data regarding mutations and copy number alterations on the summary page and the patient and sample data on the clinical data page were downloaded. All of the data were recorded in a chart for further analysis (Supplementary Dataset S1).

### Major characteristics of the *BRAF*^V600E^ and *BRAF*^non-V600E^ cohorts using univariate logistic regression analysis

The enrolled populations were divided into *BRAF*^V600E^ and *BRAF*^non-V600E^ groups. The numbers and percentages of categorical variables were calculated. Their demographic characteristics, including sex, diagnosis age, cancer type, and overall survival status, were analyzed using univariate logistic regression analysis. The odds ratios (ORs) and 95% confidence intervals (CIs) were estimated.

### Co-occurring mutations of the *BRAF*^V600E^ and *BRAF*^non-V600E^ cohorts using univariate and multivariate logistic regression analysis

The numbers and percentages of categorical variables were calculated in the *BRAF*^V600E^ and *BRAF*^non-V600E^ groups. The available data for co-occurring mutated genes in these two groups were analyzed using univariate logistic regression analysis. Thereafter, significant variables (*P* < 0.10) were analyzed using multivariate logistic regression analysis. The ORs and 95% CIs were estimated.

### Co-occurring copy number alterations in the *BRAF*^V600E^ and *BRAF*^non-V600E^ cohorts using heatmap and univariate logistic regression analysis

The available copy number alterations of the *BRAF*^V600E^ and *BRAF*^non-V600E^ cohorts were retrieved and displayed using a heatmap by Morpheus (https://software.broadinstitute.org/morpheus). The putative copy-number alterations are as follows: − 2 = homozygous deletion; − 1 = hemizygous deletion; 0 = neutral/no change; 1 = gain; 2 = high-level amplification. Univariate logistic regression analysis was used to calculate the numbers and percentages of *CDKN2A* homozygous deletion (HD) and *CDKN2B* HD. The ORs and 95% CIs were estimated.

### Crossover analysis with Kaplan–Meier survival curves and the log rank (mantel-Cox) test

The overall survival rates of the *BRAF*^V600E^ and *BRAF*^non-V600E^ cohorts were compared using Kaplan-Meier curves and the log rank (Mantel-Cox) test [23]. To exclude the benefit of *IDH1/2* on survival, we referred to the *BRAF*^V600E^ & *IDH1/2*^WT^ group as the *BRAF*^V600E^ group minus those with *IDH1/2* mutations, as well the *BRAF* ^non-V600E^ & *IDH1/2*^WT^ group as the *BRAF* ^non-V600E^ group minus those with *IDH1/2* mutations. The survival of the *BRAF*^V600E^ & *IDH1/2*^WT^ group was compared with that of the *BRAF*^non-V600E^ & *IDH1/2*^WT^ groups. There were two clusters of mutations, one in the glycine-rich loop (referred to as the G-loop) and the other in the activation segment. To evaluate the effect of the mutation site on survival, we defined two subgroups in the *BRAF*^non-V600E^ & *IDH1/2*^WT^ group. One subgroup was the *BRAF*^non-V600E^ & *IDH1/2*^WT^ group with the mutation site in the G-loop, and the other subgroup was the *BRAF*^non-V600E^ & *IDH1/2*^WT^ group with the mutation site in the activation segment. The *BRAF*^V600E^ & *IDH1/2*^WT^ group was compared with those two subgroups. Furthermore, the G-loop *BRAF*^non-V600E^ & *IDH1/2*^WT^ subgroup was compared with the remaining patients in the *BRAF*^non-V600E^ & *IDH1/2*^WT^ group.

### Statistical analysis

Major characteristics, co-occurring mutations and copy number alterations of the *BRAF*^V600E^ and *BRAF*^non-V600E^ cohorts were analyzed using univariate logistic regression analysis. Significant variables (*P* < 0.10) of co-occurring mutations of the *BRAF*^V600E^ and *BRAF*^non-V600E^ cohorts were analyzed using multivariate logistic regression analysis. Kaplan-Meier curves were generated for glioma patients with *BRAF* mutations and were compared using the log-rank (Mantel-Cox) test. A *P* value < 0.05 was considered statistically significant.

## Results

### Data enrollment in the study

**Fig. 1:**
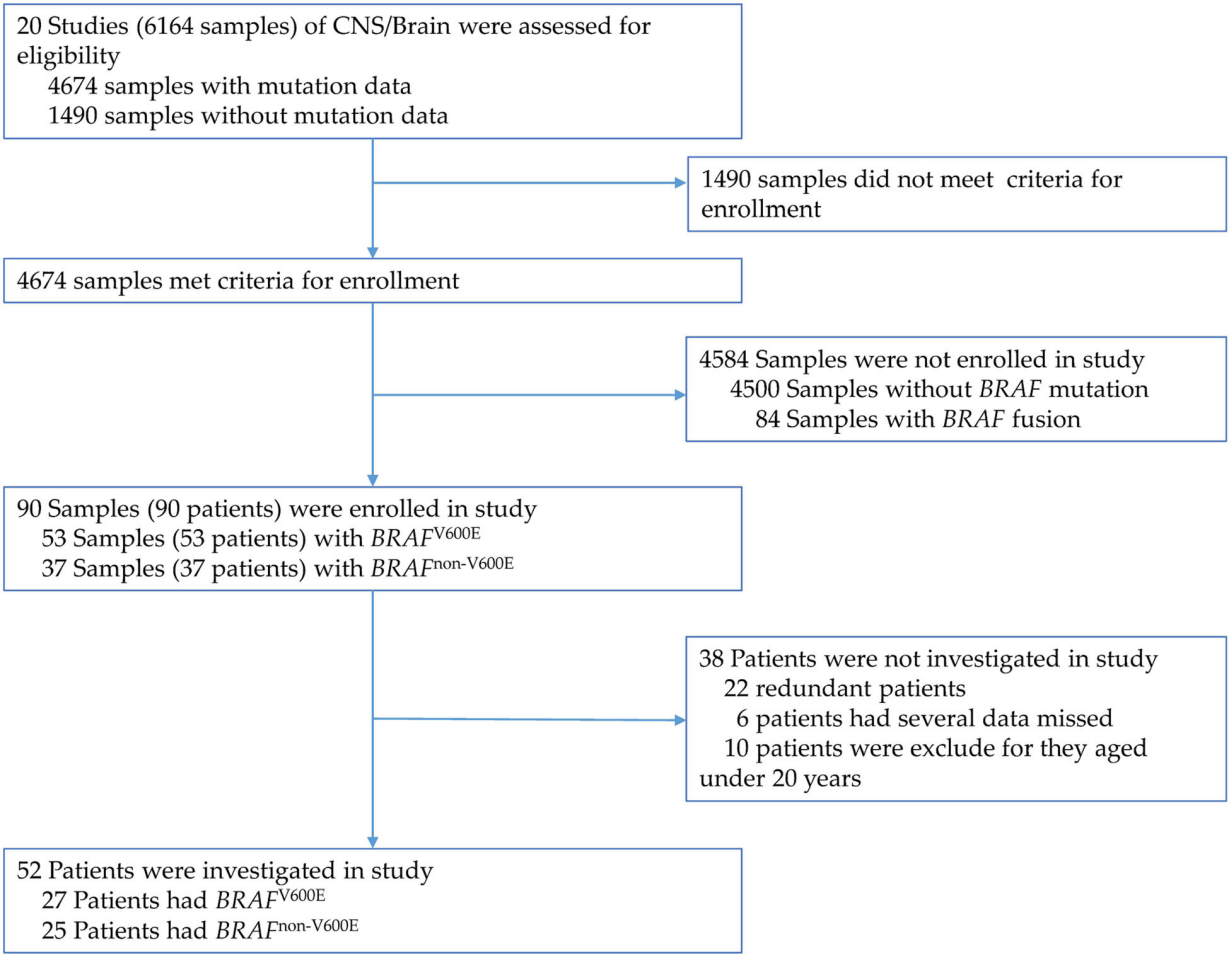
The scheme of the enrollment and investigation of data. In all 20 CNS/Brain studies, including 6164 samples, 90 patients with *BRAF*^V600E^ or *BRAF*^non-V600E^ were enrolled; 52 nonredundant patients displayed major patient characteristics, including sex, age, cancer type detailed, co-occurring mutations, and copy number alteration genes, and were enrolled for further analysis

In all 20 CNS/brain studies (6164 samples), 4674 samples with mutation data were queried; 90 samples (90 patients) with *BRAF* mutations, including 53 samples (53 patients) with *BRAF*^V600E^ and 37 samples (37 patients) with *BRAF*^non-V600E^, are shown in Table 1. The cancer types of 20 CNS/brain studies included diffuse glioma, glioblastoma, oligodendroglioma, embryonal tumor, encapsulated glioma, and miscellaneous neuroepithelial tumor. The scheme for the final enrolled and investigated data is shown in Fig. 1. Ninety patients with *BRAF*^V600E^ or *BRAF*^non-V600E^ were enrolled in this study, and data from 52 nonredundant patients were investigated. The integrated data of their major patient characteristics, including sex, age, diagnosis age, cancer type, data of co-occurring mutations, copy number alterations, and overall survival time and status, were collected for further analysis.

**Table 1:** The CNS/brain projects of TCGA database enrolled in the study retrieved by cBioPortal

Project	All Samples	Samples with mutation data	Samples with ***BRAF***^**V600E**^	Samples with ***BRAF***^**non-V600E**^	References
Diffuse Glioma					
Brain Lower Grade Glioma (TCGA, Firehose Legacy)	530	286	1	1	https://www.cancer.gov
Brain Lower Grade Glioma (TCGA, PanCancer Atlas)	514	512	1	2	[24–29]
Glioma (MSK, Nature 2019)	91	91	2	1	https://www.cancer.gov
Glioma (MSKCC, Clin Cancer Res 2019)	1004	1004	22	19	[30]
Low-Grade Gliomas (UCSF, Science 2014)	61	61	2	0	[31]
Merged Cohort of LGG and GBM (TCGA, Cell 2016)	1102	812	5	2	[32]
GLIOBLASTOMA					
Brain Tumor PDXs (Mayo Clinic, 2019)	95	83	2	1	https://www.cbioportal.org
Glioblastoma (Columbia, Nat Med. 2019)	42	32	1	1	[33]
Glioblastoma (TCGA, Cell 2013)	543	257	3	0	[34]
Glioblastoma (TCGA, Nature 2008)	206	91	0	0	[35]
Glioblastoma Multiforme (TCGA, Firehose Legacy)	604	290	5	1	https://www.cancer.gov
Glioblastoma Multiforme (TCGA, PanCancer Atlas)	592	397	5	3	[24–29, 36]
OLIGODENDROGLIOMA					
Anaplastic Oligodendroglioma and Anaplastic Oligogastrocytoma (MSKCC, Neuro Oncol 2017)	22	22	0	0	[37]
Embryonal Tumor					
MEDULLOBLASTOMA					
Medulloblastoma (Broad, Nature 2012)	92	92	0	0	[38]
Medulloblastoma (ICGC, Nature 2012)	125	125	0	0	[39]
Medulloblastoma (PCGP, Nature 2012)	37	37	0	0	[40]
Medulloblastoma (Sickkids, Nature 2016)	46	46	0	1	[41]
Encaspulated Glioma					
PILOCYTIC ASTROCYTOMA					
Pilocytic Astrocytoma (ICGC, Nature Genetics 2013)	96	96	4	3	[42]
Miscellaneous Neuroepithelial Tumor					
Pheochromocytoma and Paraganglioma (TCGA, Cell 2017)	178	178	0	1	[43]
Pheochromocytoma and Paraganglioma (TCGA, Firehose Legacy)	184	162	0	1	https://www.cancer.gov

### Major characteristics of the cohorts with *BRAF*^V600E^ and *BRAF*^non-V600E^

The study populations were divided into two groups, *BRAF*^V600E^ and *BRAF*^non-V600E^. The major demographic characteristics and clinical data of the two groups are summarized in Table 2. The patients’ ages ranged from 20 to 85 years and were divided into early adulthood, midlife, mature adulthood, and late adulthood (aged 20–35, 35–50, 50–80, and > 80 years, respectively). The two groups had comparable proportions of male patients, diagnosis age, cancer type, and overall survival status. Glioblastoma multiform was the most common cancer type in both cohorts (74.07% vs. 56.00%; *P* = 0.175; Table 2).

**Table 2:** The major characteristics of cohorts including *BRAF*^V600E^ and *BRAF*^non-V600E^

Variables	***BRAF***^**V600E**^ (***n*** = 27)		***BRAF***^**non-V600E**^ (***n*** = 25)		Univariate analysis		
	Number	%	Number	%	Odds Ratio	95% Confidence Interval	***P*** Value
Male	16	59.26	18	72.00	0.566	0.177–1.809	0.337
Diagnosis Age							
Ages 20–35	9	33.33	6	24.00	1.583	0.469–5.350	0.459
Ages 36–50	9	33.33	8	32.00	1.062	0.333–3.390	0.918
Ages 51–80	7	25.93	11	44.00	0.445	0.139–1.433	0.175
Age 80+	2	7.41	0	0.00	1,615,474,843	0.000-	0.999
Cancer type detailed							
Glioblastoma Multiform	20	74.07	14	56.00	2.245	0.698–7.219	0.175
Astrocytoma	3	11.11	6	24.00	0.396	0.087–1.794	0.229
Oligoastrocytoma	1	3.70	0	0.00	1,553,341,195	0.000-	1.000
Oligodendroglioma	0	0.00	3	12.00	0.000	0.000-	0.999
Gliosarcoma	0	0.00	2	8.00	0.000	0.000-	0.999
Other glioma	3	11.11	0	0.00	1,682,786,295	0.000-	0.999
Overall survival status							
Deceased	14	51.85	11	44.00	1.371	0.460–4.087	0.572

### Co-occurring mutations of the *BRAF*^V600E^ and *BRAF*^non-V600E^ cohorts using univariate and multivariate logistic regression analysis

Available co-occurring gene mutations of *the BRAF*^V600E^ and *BRAF*^non-V600E^ cohorts were retrieved, and differences between the two groups were compared; the results are summarized in Table 3. The mutation frequencies of *KRAS*, *HRAS*, *RAF1*, *MAP 3 K1*, *MAP 2 K1*, *MAP 2 K2*, *MAP 2 K4*, *MDM2*, *MDM4*, *CDKN2A*, and *CDKN2B* were comparable between the two groups. In contrast, the *BRAF*^non-V600E^ group exhibited a significantly higher mutation frequency of *TP53* (56.00% vs. 7.41%; *P* = 0.001), *IDH1*/*2* (36.00% vs. 3.70%; *P* = 0.015), and *ATRX* (32.00% vs. 7.41%; *P* = 0.037) than the *BRAF*^V600E^ group. The variables with *P* < 0.10 were analyzed using multivariate logistic regression analysis, and the *BRAF*^non-V600E^ group exhibited a significantly higher *TP53* mutation frequency (56.00% vs. 7.41%; *P* = 0.031) than the *BRAF*^V600E^ group (Table 3).

**Table 3:** The co-occurred mutations of BRAF^V600E^ and BRAF^non-V600E^ cohort using univariate and multivariate logistics regression analysis

Gene	***BRAF***^**V600E**^ (***n*** = 27)		***BRAF***^**non-V600E**^ (***n*** = 25)		Univariate analysis			Multivariate analysis		
	Number	%	Number	%	Odds Ratio	95% Confidence Interval	***P*** Value	Odds Ratio	95% Confidence Interval	***P*** Value
*KRAS*	0	0.00	1	4.00	1,817,409,198	0.000-	1.000			
*HRAS*	0	0.00	1	4.00	1,817,409,198	0.000-	1.000			
*RAF1*	0	0.00	2	8.00	1,896,426,989	0.000-	0.999			
*MAP 3 K1*	1	3.70	6	24.00	8.211	0.911–73.959	0.060			
*MAP 2 K1*	0	0.00	2	8.00	1,896,426,989	0.000-	0.999			
*MAP 2 K2*	0	0.00	4	16.00	2,077,039,084	0.000-	0.999			
*MAP 2 K4*	0	0.00	2	8.00	1,896,426,989	0.000-	0.999			
*TP53*	2	7.41	14	56.00	15.909	3.078–82.224	0.001	12.186	1.251–118.721	0.031
*MDM2*	1	3.70	3	12.00	3.545	0.344–36.561	0.298			
*MDM4*	0	0.00	4	16.00	2,077,039,084	0.000-	0.999			
*CDKN2A*	0	0.00	3	12.00	1,982,628,216	0.000-	0.999			
*CDKN2B*	0	0.00	1	4.00	1,817,409,198	0.000-	1.000			
*IDH1/2*	1	3.70	9	36.00	14.625	1.690–126.537	0.015	5.498	0.512–59.020	0.159
*ATRX*	2	7.41	8	32.00	5.882	1.110–31.170	0.037	0.665	0.048–9.188	0.761

### Co-occurring copy number alteration in the *BRAF*^V600E^ and *BRAF*^non-V600E^ cohorts using heatmap and univariate logistic regression analysis

**Fig. 2:**
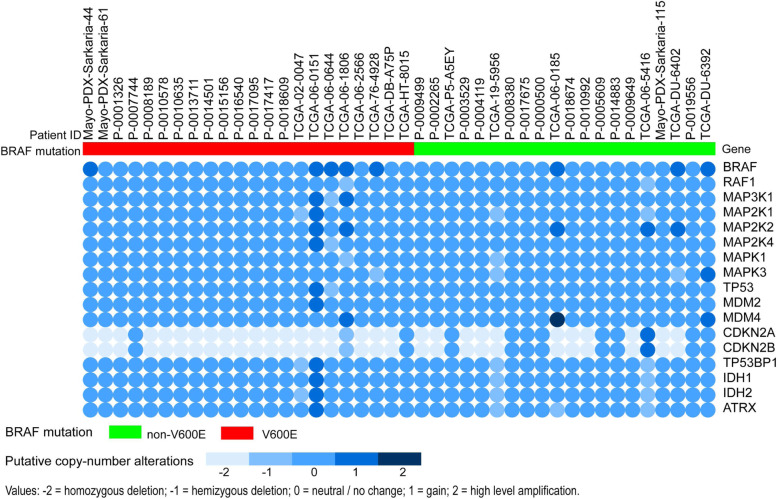
The co-occurring copy number alterations of the *BRAF*^V600E^ cohort and *BRAF*^non-V600E^ cohort using a heatmap. The cohorts of *BRAF*^V600E^ (red) or *BRAF*^non-V600E^ (green) are shown, and putative copy-number alterations change from light to dark with value enhancement

There were no available copy number data for five patients with *BRAF*^V600E^ and five patients with *BRAF*^non-V600E^. The copy number alterations of the available co-occurring genes included *BRAF*, *RAF1*, *MAP 3 K1*, *MAP 2 K1*, *MAP 2 K2*, *MAP 2 K4*, *MAPK1*, *MAPK3*, *TP53*, *MDM2*, *MDM4*, *TP53BP1*, *IDH1*, *IDH2*, *ATRX*, *CDKN2A*, and *CDKN2B*. The HD copy number was frequently retrieved for these two genes, including *CDKN2A* and *CDKN2B* (Fig. 2), and the HD of both *CDKN2A* (77.27.00% vs. 60.00%; *P* = 0.032) and *CDKN2B* (77.27.00% vs. 60.00%; *P* = 0.032) was more frequent in the *BRAF*^V600E^ cohort than in the *BRAF*^non-V600E^ cohort (Table 4).

**Table 4:** *CDKN2A*/*2B* HD of *BRAF*^V600E^ and *BRAF*^non-V600E^ cohort using univariate logistics regression analysis

Variables	***BRAF***^**V600E**^ (***n*** = 22)		***BRAF***^**non-V600E**^ (***n*** = 20)		Univariate analysis		
	Number	%	Number	%	Odds Ratio	95% Confidence Interval	***P*** Value
*CDKN2A*	17	77.27	12	60.00	0.193	0.043–0.867	0.032
*CDKN2B*	17	77.27	12	60.00	0.193	0.043–0.867	0.032

### Crossover analysis using Kaplan–Meier survival curves and the log-rank (mantel-Cox) test

**Fig. 3:**
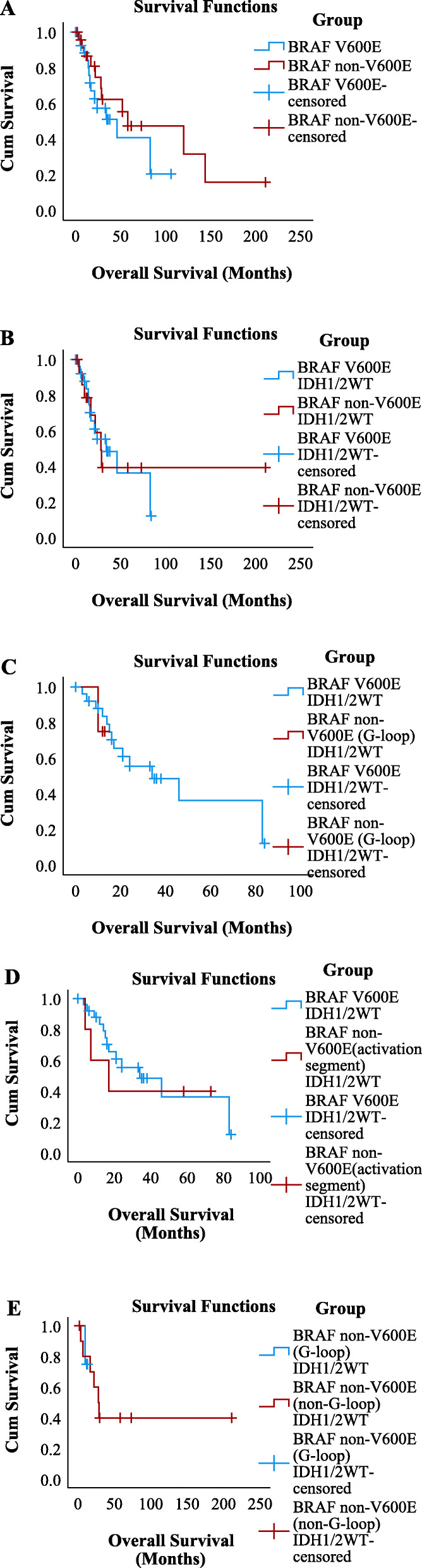
Crossover analysis with Kaplan–Meier survival curves and the log-rank (Mantel-Cox) test. **a** *BRAF*^V600E^ vs. *BRAF*^non-V600E^ (51.394 vs. 89.958, Chi-Square 1.130, *P* = 0.288); **b** *BRAF*^V600E^ & *IDH1/2*^WT^ vs. *BRAF*^non-V600E^ & *IDH1/2*^WT^ (44.500 vs. 93.821, Chi-Square 0.007, *P* = 0.935); **c** *BRAF*^V600E^ & *IDH1/2*^WT^ vs. *BRAF*^non-V600E^ & *IDH1/2*^WT^ with mutations in G-loop (44.500 vs. 12.250, Chi-Square 0.122, *P* = 0.727); **d** *BRAF*^V600E^ & *IDH1/2*^WT^ vs. *BRAF*^non-V600E^ & *IDH1/2*^WT^ with mutations in activation segment (44.500 vs. 34.800, Chi-Square 0.145, *P* = 0.703); **e** *BRAF*^non-V600E^ & *IDH1/2*^WT^ with mutations in G-loop vs. the rest *BRAF*^non-V600E^ & *IDH1/2*^WT^ patients (12.250 vs. 95.100, Chi-Square 0.008, *P* = 0.927)

Crossover Kaplan–Meier survival curves and the log-rank (Mantel-Cox) test were performed to explore the difference between the overall survival of glioma patients with *BRAF*^V600E^ and *BRAF*^non-V600E^. The estimated mean survival time was 51.394 months for patients with *BRAF*^V600E^, 89.958 months for patients with *BRAF*^non-V600E^, 44.500 months for patients with *BRAF*^V600E^ & *IDH1/2*^WT^, and 93.821 months for patients with *BRAF*^non-V600E^ & *IDH1/2*^WT^. There was no difference between the survival of *BRAF*^V600E^ and *BRAF*^non-V600E^ (51.394 vs. 89.958, chi-square 1.130, *P* = 0.288). In addition, there was no difference between the survival of *BRAF*^V600E^ & *IDH1/2*^WT^ and *BRAF*^non-V600E^ & *IDH1/2*^WT^ (44.500 vs. 93.821, chi-square 0.007, *P* = 0.935), which excluded the survival benefit of *IDH1/2*. We also evaluated the survival of *BRAF*^non-V600E^ & *IDH1/2*^WT^ with mutations in the G-loop and activation segment. The estimated survival time of these two subgroups was 12.250 months for patients with *BRAF*^non-V600E^ & *IDH1/2*^WT^ with mutations in the G-loop and 34.800 months for patients with *BRAF*^non-V600E^ & *IDH1/2*^WT^ with mutations in the activation segment. In addition, there was no difference between the *BRAF*^V600E^ & *IDH1/2*^WT^ cohorts and those of the *BRAF*^non-V600E^ & *IDH1/2*^WT^ cohorts. As shown below, *BRAF*^V600E^ & *IDH1/2*^WT^ vs. *BRAF*^non-V600E^ & *IDH1/2*^WT^ had mutations in the G-loop (44.500 vs. 12.250, chi-squared 0.122, *P* = 0.727), and *BRAF*^V600E^ & *IDH1/2*^WT^ vs. *BRAF*^non-V600E^ & *IDH1/2*^WT^ had mutations in the activation segment (44.500 vs. 34.800, chi-square 0.145, *P* = 0.703). Since the estimated mean survival of *BRAF*^non-V600E^ & *IDH1/2*^WT^ with mutations in the G-loop was the shortest, we compared the *BRAF*^non-V600E^ & *IDH1/2*^WT^ with mutations in the G-loop with the remaining *BRAF*^non-V600E^ & *IDH1/2*^WT^ patients. There was no difference between them (12.250 vs. 95.100, chi-square 0.008, *P* = 0.927) (Fig. 3). The numbers at risk of Kaplan–Meier survival curves were shown in Supplementary Dataset S2.

## Discussion

*BRAF* mutations critically affect cancer growth and progression and are supposed to be a founder event for mutations occurring early in the initiation process of cancer. However, *BRAF* mutations must cooperate with other mechanisms for a fully cancerous state, as they are insufficient to induce cancer alone [5]. *BRAF*^V600E^ has been the mutation of interest in previous studies on glioma, especially in pediatric glioma patients, for the available molecule-targeted drugs. However, various *BRAF*^non-V600E^ cells exert different activation effects on the MAPK pathway. The exact impact on the clinical prognosis and possible molecular mechanism of associated co-occurring genes with mutations or copy number alterations co-occurring with *BRAF* mutations remains unclear in adult glioma patients. In this study, the available data of patients with *BRAF*^non-V600E^ and *BRAF*^V600E^ in the TCGA CNS/brain database were investigated to determine the possible mechanisms of *BRAF* gene mutations in adult glioma patients.

Our data indicated that in adult glioma patients with *BRAF* mutations, including both *BRAF*^non-V600E^ and *BRAF*^V600E^ cohorts, glioblastoma multiform was the most common cancer type. A previous study showed that all *BRAF*^V600E^ glioblastomas were primary tumors in both pediatric and adult patients [44]. Tabouret et al. [20] reported a case the co-occurrence of both *IDH1* mutation and *BRAF*^V600E^ although those two mutations are mutually exclusive in glial tumor. The available co-occurring mutated genes in the MAPK and p53 pathways showed that mutated genes frequently co-occurred in the *BRAF*^non-V600E^ cohort, and there were more *TP53*, *IDH1/2*, and *ATRX* mutations in *BRAF*^non-V600E^ than in *BRAF*^V600E^. Lai et al. [45] found that a *TP53* point mutation at position 273 (Arg to Cys) was more common than *IDH1* mutations at position 132 (Arg to His). They hypothesized that the *TP53* mutation (C → T) occurred in the nontranscribed strand, while the *IDH1* mutation existed in the transcribed strand, which is a strand asymmetry pattern [46]. Another study indicated that *IDH1/2* mutations represent early events in brain tumor formation [47]. Liu et al. [48] found that *ATRX* alterations correlated with mutations in *IDH1/2* and *TP53* in glioma of all grades. It has been reported that *ATRX* deletions/mutations are correlated with *TP53* and *IDH1* mutations [49, 50]. Somatic *TP53*, *ATRX*, and *IDH1/2* mutations have been found in adult LGGs [51]. *ATRX* mutations are detected in adult diffuse gliomas and astrocytomas harboring both *TP53* and *IDH1/2*. The co-occurrence of these three mutated genes, including *TP53*, *IDH1/2,* and *ATRX,* facilitates the growth of an adult diffuse astrocytoma subgroup [48]. All of the studies above indicate that *ATRX* mutations frequently overlap with *IDH1/2* and *TP53* mutations. In the present study, we also found the co-occurrence of these three mutations, which were frequently detected in the *BRAF*^non-V600E^ cohort but not in the *BRAF*^V600E^ cohort. Our findings indicated that in adult glioma patients, a possible correlation between *BRAF*^non-V600E^ and these three common mutations simultaneously occurred in glioma. Multivariate logistic regression revealed that *TP53* was an independent risk factor in the *BRAF*^non-V600E^ cohort vs. the *BRAF*^V600E^ group. Our data demonstrated a correlation between *BRAF*^non-V600E^ and *TP53* mutations in adult glioma patients.

Previous findings have shown that active *Ras* can induce heterodimerization of *BRAF* and *RAF1* [52] and that this event may be critical for *RAF1* activation [53]. *RAF1* directly regulates cell apoptosis, which does not depend on MAPK signaling [54, 55], but occurs through direct interaction with *Bcl-2* [54]. *TP53* can regulate *Bcl-2* by suppressing *Bcl-2* transcription [56]. We proposed that the *BRAF*^non-V600E^ mutation might activate the *BRAF-RAF1* heterodimer, which shows antiapoptotic properties via the activation of Bcl-2 through *RAF1* phosphorylation. Mutant *TP53*, which is frequently accompanied by *IDH1/2* mutation by a strand asymmetry mechanism, fails to regulate *Bcl-2*. Therefore, with both activated *RAF1* and mutated *TP53*, an enhanced antiapoptotic effect, which promotes cancer growth, might be predicted.

Compared to BRAF fusions, *BRAF*^V600E^ tends to be more aggressive, more likely to be associated with *CDKN2A/B* deletions, and can transform cancers into higher-grade tumors [57, 58]. Our data showed that *CDKN2A* and *CDKN2B* HDs were more frequent in the *BRAF*^V600E^ cohort than in the *BRAF*^non-V600E^ cohort. Concomitant *CDKN2A* and *CDKN2B* HDs could be detected in patients with glioblastoma multiform cancer, astrocytoma, and gliosarcoma. A previous report indicated that five of seven pediatric grade II–IV astrocytomas with *BRAF*^V600E^ had concomitant *CDKN2A* HD [59] and *CDKN2A* deletions combined with *BRAF*^V600E^ alterations, constituting a subgroup of secondary high-grade gliomas [60]. We found that in adult glioma patients, *BRAF*^V600E^ and *BRAF*^non-V600E^ frequently co-occurred with *CDKN2A* HDs combined with *CDKN2B* HDs, especially in patients with *BRAF*^V600E^. Except for astrocytoma, glioblastoma multiform cancer was the most common cancer type with these combined alterations. Robinson et al. [61] indicated that activated Akt or Ink4a/ARF deletions are necessary for high-grade brain neoplasms with *BRAF* mutations in a Cre/lox animal model. Our results showed the possible synergy of *CDKN2A* and *CDKN2B* HDs with *BRAF* mutations, especially in adult glioma patients with *BRAF*^V600E^ and *BRAF*^non-V600E^.

*BRAF*^V600E^ reportedly enhances *BRAF* kinase activity 500-fold [62]. According to its kinase viability, *BRAF*^non-V600E^ mutations can be classified into three groups: high activity (130–700 times), intermediate activity (1.3–64 times), and impaired activity (30–80%) [16]. Theoretically, the higher the *BRAF* kinase activity, the worse the prognosis. To clarify whether there is a difference between *BRAF*^V600E^ and *BRAF*^non-V600E^, we compared the overall survival of these two cohorts, and no statistical significance was found.

In addition, the status of *IDH* mutations in glioblastomas definitely influences the prognosis of patients with glioblastomas; therefore, *IDH*-wildtype glioblastomas are defined as primary tumors, while *IDH*-mutant glioblastomas are classified as secondary tumors [63]. To exclude the benefit of *IDH* mutations on survival, we compared the *BRAF*^V600E^ & *IDH1/2*^WT^ and *BRAF*^non-V600E^ & *IDH1/2*^WT^ cohorts, and no difference was detected. The positions of the G-loop and the activation segment are 458–470 aa and 577–622 aa in BRAF, respectively [64]. Most *BRAF*^non-V600E^ mutations exist in the G-loop and the activation segment [16, 64]; therefore, we selected the two cohorts as *BRAF*^non-V600E^ & *IDH1/2*^WT^ with mutations in the G-loop and activation segment. We compared them with *BRAF*^V600E^ & *IDH1/2*^WT^, and no difference was found between the *BRAF*^V600E^ & *IDH1/2*^WT^ cohorts and those of the *BRAF*^non-V600E^ & *IDH1/2*^WT^ cohorts. Furthermore, we compared *BRAF*^non-V600E^ & *IDH1/2*^WT^ with mutations in the G-loop with the remaining *BRAF*^non-V600E^ & *IDH1/2*^WT^ patients and found no difference between them. Although there was no statistical significance, the estimated mean survival of *BRAF*^non-V600E^ & *IDH1/2*^WT^ with mutations in the G-loop was the shortest in all cohorts. We propose that a larger sample is necessary for confirmation of this finding. Our data indicated that the *BRAF*^non-V600E^ cohort had no survival advantage from co-occurrence with *IDH* mutations compared with the *BRAF*^non-V600E^ cohort of adult patients with glioma.

### Limitations

Because the *BRAF*^V600E^ mutation is rare in adult glioma, there were few patients in both cohorts retrieved from the publicly available data (cBioPortal). In this study, while their apparent survival times were substantially different, they were not significantly different. To prove the mechanism by which *BRAF* mutations promote cancer growth via an enhanced antiapoptotic effect of *Bcl-2*, further study using appropriate clinical tissue samples or animal models are necessary.

## Conclusions

In conclusion, we found that in adult patients with gliomas, *BRAF*^non-V600E^, rather than *BRAF*^V600E^, frequently co-occurs with *TP53*, *IDH1/2*, and *ATRX* mutations. Both *BRAF*^non-V600E^ and *BRAF*^V600E^ frequently overlapped with *CDKN2A/2B* HDs, whereas there were no significant differences between the two cohorts. Although there were significant differences in co-occurring gene mutations and copy number alterations, no difference was found in survival between cohorts of *BRAF*^non-V600E^ and *BRAF*^V600E^ with and without IDH1/2 favorable effects on survival. We also found that the estimated mean survival of *BRAF*^non-V600E^ & *IDH1/2*^WT^ with mutations in the G-loop was the shortest; however, no difference was observed between that cohort and other cohorts. Due to the poor available mRNA and protein data in the TCGA database we retrieved in this study, no expression data were evaluated. More clinical data or models are necessary to elucidate the mechanism involved in *BRAF*^non-V600E^-associated glioma in the future.

## Supplementary Information

**Additional file 1.**

**Additional file 2.**

## Data Availability

All data generated or analyzed during this study are included in this published article and its supplementary information files.

## Abbreviations

HD: Homozygous deletion
*BRAF*: v-raf murine sarcoma viral oncogene homolog B1
LGGs: Low-grade gliomas
TCGA: The Cancer Genome Atlas
ORs: Odds ratios
Cis: Confidence intervals
G-loop: Glycine-rich loop
MAPK: Mitogen-activated protein kinase
